# Supplementary material for: Efficient Transformation of Somatic Embryos and Regeneration of Cork Oak Plantlets with a Gene (CsTL1) Encoding a Chestnut Thaumatin-Like Protein
Source: Int J Mol Sci. 2021 Feb 10;22(4):1757. doi: 10.3390/ijms22041757 (PMC7916472; doi:10.3390/ijms22041757)
Supplement: Supplementary file 1 [file ijms-22-01757-s001.pdf]

International Journal of *Molecular Sciences*

MDPI

Article

Efficient Transformation of Somatic Embryos and Regeneration of Cork Oak Plantlets with A Gene (*CsTL1*) Encoding a Chestnut Thaumatin-Like Protein

Vanesa Cano [1], Mª Teresa Martínez [1], José Luis Couselo [2,3], Elena Varas [3], Francisco Javier Vieitez [1] and Elena Corredoira [1,*]

1 Instituto de Investigaciones Agrobiológicas de Galicia (IIAG), Avd Vigo s/n, 15705 Santiago de Compostela, Spain; vanesa.cano.lazaro@iiag.csic.es (V.C.); temar@iiag.csic.es (Mª.T.M.); jvieitez@iiag.csic.es (F.J.V.)
2 Universidad de Vigo, Edif. De Ciencias Experimentales, Bloque A, Campus Universitario, 36310 Vigo, Spain; jlcouselo@semillasfito.com (J.L.C.)
3 Estación Fitopatolóxica do Areeiro (EFA), Subida a la Robleda s/n, 36153 Pontevedra, Spain; elenav@promiva.es (E.V.)
* Correspondence: elenac@iiag.csic.es

Supplementary material:

Online Resource 1. Cryopreservation of transgenic and non-transgenic (wt) cork oak lines.

Online Resource 1A. A. Embryo survival and embryo recovery rates in cryopreserved cork oak somatic embryos of transgenic and non-transgenic (wt) lines after preculture on sucrose medium, exposure to PVS2 solution for 60 min at 0ºC and subsequent immersion in liquid nitrogen.

EMBRYOGENIC LINE	SURVIVAL (%)	EMBRYO RECOVERY (%)
**ALM6-wt**	63.3 ± 7.0	60.0 ± 5.8
ALM6-tau 1	86.7 ± 1.9	80.0 ± 0.0
ALM6-tau 6	96.7± 1.9	90.0 ± 0.0
ALM6-tau 12	93.3 ± 1.9	86.7 ± 5.1
**ALM80-wt**	90.0 ± 5.8	83.3 ± 3.9
ALM80-tau 13	96.7± 1.9	76.7 ± 1.9
ALM80-tau 19	96.7 ± 1.9	93.3 ± 1.9
ALM80-tau 20	80.0 ± 0.0	76.7 ± 1,9
**TGR3-wt**	66.7 ± 5.1	66.7 ± 5.1
TGR3-tau 2	83.3 ±4.7	80.0 ± 8.2
TGR3-tau 4	63.3 ± 18.9	53.3 ± 12.5
TGR3-tau 5	90.0 ± 3.4	90.0 ± 3.4
TGR3-tau 6	100.0 ± 0.0	100.0 ± 0.0
TGR3-tau 9	90.0 ± 8.2	80.0 ± 0.0
TGR3-tau 18	100.0 ± 0.0	100.0 ± 0.0
TGR3-tau 21	93.3 ± 1.9	93.3 ± 1.9
TGR3-tau 23	93.3 ± 4.7	83.3 ± 4.7
TGR3-tau 34	76.7 ± 17.0	66.7 ± 9.4
TGR3-tau 36	90.0 ± 10.0	75.0 ± 25.0
TGR3-tau 42	90.0 ± 14.1	86.7 ± 18.9
TGR3-tau 45	96.7 ± 4.7	70.0 ± 8.2

Each value represents the mean ± standard error (SE) of 3 replicates (10 explants each).

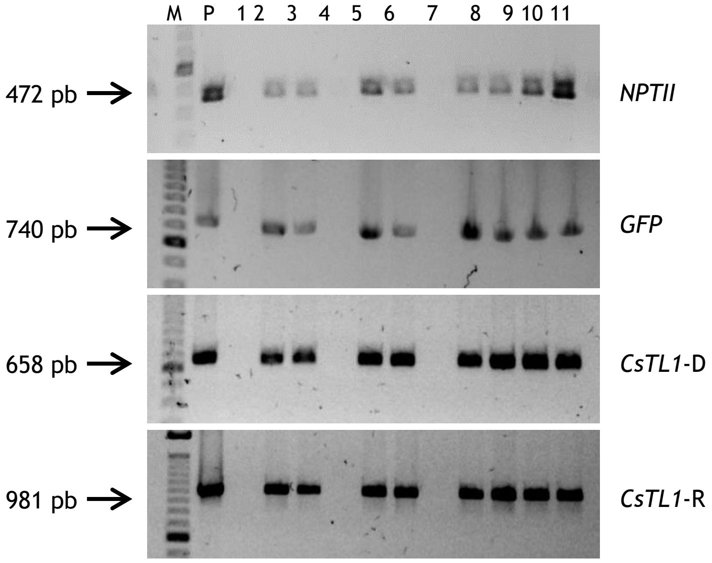

Online Resource 1B. PCR analysis of transgenic and non-transgenic (wt) lines after the cryopreservation process. M: molecular weight marker; P: plasmid (positive control); lanes 1-3: ALM6 non-transgenic (1) and transformed lines (2, 3); lanes 4-6: ALM80 non-transgenic (4) and transformed lines (5, 6); lanes 7-11: TGR3 non-transgenic (7) and transformed lines (8-11).

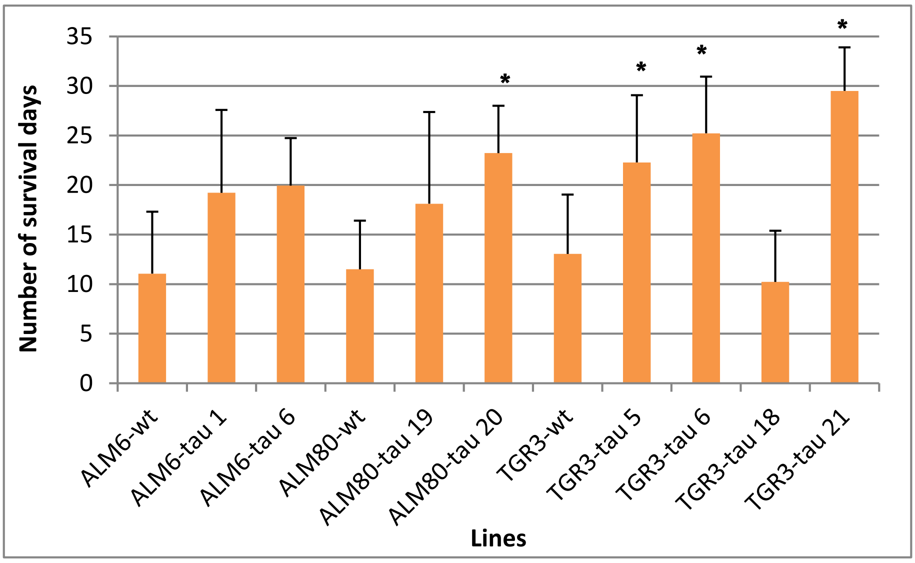

Online Resource 2. Number of days of survival of the transgenic and non-transgenic (wt) cork oak lines infected with *P. cinnamomi* and incubated for a period of 31 days. A total of 18 plants were evaluated for each line. Data analyzed by Kruskal-Wallis (p≤0.05). The error bars represent the standard errors. Asterisks indicate statistical significance (p≤0.05) of the lines with more days of survival.

Online Resource 3. Primers and PCR conditions used in the study.

Gene	Name	Primer sequence (5′-3′)	PCR conditions	Fragment amplified (bp)	Purpose
NPTII	NPTII-F	GTCATCTCACCTTGCTCCTGCC	35 cycles: 94ºC x 30s 60ºC x 30s 72ºC x 42s	472	PCR analysis
	NPTII-R	AAGAAGGCGATAGAAGCGA			
EGFP	EGFP-F	CACCGGGGTGGTGCCCAT	40 cycles:	740	PCR analysis

	EGFP-R	CTAGTGGATCCCCCGGGC	94ºC x 15s<br>60ºC x 30s<br>72ºC x 1min		
CsTL1-Forward[1]	CsTL1-F-F	AGGTCACTGGATTTTGGT	40 cycles:<br>94ºC x 15s<br>60ºC x 30s<br>72ºC x 1min	981	PCR analysis
	CsTL1-F-R	CACCATGATGAAAACCCTG			
CsTL1-Reverse[2]	CsTL1-R-F	GGTAAGGCCGTAGAGT	40 cycles:<br>94ºC x 15s<br>60ºC x 30s<br>72ºC x 1min	658	PCR analysis
	CsTL1-R-R	GATCTAACAGAACTCGCC			
CsTL1	CsTL1-Fq	GTTCAAGCTCCATGGAAAGG	40 cycles:<br>95ºC x15s<br>60ºC x 1min	-	qPCR analysis of *CsTL1*
	CsTL1-Rq	ACCTGACCGGTGCTACAATC			
TUB	QpTUBqF	CTCGTGCTGTTCTCATGGATCT	40 cycles:<br>95ºC x15s<br>60ºC x 1min	-	Reference gene for qPCR
	QpTUBqR	TGGCCGAAAACGAAGTTGTC			
ACT	QsACT-F	GCCCCACGAGCTGTGTTC	40 cycles:<br>95ºC x15s<br>60ºC x 1min	-	Reference gene for qPCR
	QsACT-R	TCTGGCCCATTCCAACCA			
EF	QsEF-F	TTGTGCCGTCCTCATTATTGACT	40 cycles:<br>95ºC x15s<br>60ºC x 1min	-	Reference gene for qPCR
	QsEF-R	TCACGGGTCTGACCATCCTT			

CsTL1 gene was confirmed by PCR in both transcriptional senses using the specific primers CsTL1-Forward and CsTL1-Reverse. [1]This fragment includes T-35S region. [2]This fragment includes p35S region. F: forward; R: reverse;*TUB*: tubulin; *ACT*: actin; *EF*: elongation factor.
